# Diabetic Foot Syndrome: A Paradigm of Complexity and a Call for Comprehensive Ownership by the Diabetologists

**DOI:** 10.1002/dmrr.70234

**Published:** 2026-09-23

**Authors:** Vittorio Oteri, Luca Dalla Paola

**Affiliations:** ^1^ Diabetic Foot Department Maria Cecilia Hospital GVM Care & Research Cotignola Italy; ^2^ Department of Clinical and Experimental Medicine Endocrinology Section Garibaldi‐Nesima Hospital University of Catania Catania Italy

**Keywords:** cardiovascular risk, diabetic foot syndrome, healthcare fragmentation, medical education, multidisciplinary team, orthoplastic reconstruction

## Conflicts of Interest

The authors declare no conflicts of interest.

To the Editor,

We are writing to highlight a critical paradox within contemporary diabetology: the systematic underestimation of diabetic foot syndrome (DFS). Despite its extreme complexity, this syndrome is frequently overlooked, causing clinicians to forfeit an unparalleled professional opportunity, whereas patients are still lacking the high‐level comprehensive care they deserve; this phenomenon is visibly mirrored in countries like Italy, where structural fragmentation often impacts care equity [1].

This gap in diabetes care is clinically unjustified nowadays. People with DFS are at extreme cardiovascular, renal, metabolic, and infective risk; their short‐term mortality risk is so staggering that the disease carries a burden akin to advanced oncology [2]. This is a condition that gives the illusion of chronicity but effectively ‘metastasises’ and manifests in an acute manner through lower limb complications and systemic failure. Such a crisis cannot be managed with fragmented, reactive care that arrives too late; it demands a proactive approach orchestrated by a clear team leader, to mitigate lower limb amputations and mortality risk of people with DFS [3].

In this scenario, we should move away from the outdated perception that diabetic foot care is a mere technical hyper‐specialisation; in reality, it demands the multifaceted, holistic management of complex patients, and diabetologists possess the comprehensive medical background required to address this syndrome.

In order to fine‐tune these clinical competencies, specific interdisciplinary management workshops led by specialists representing multiple disciplines (diabetology and endocrinology, cardiology, nephrology, infectious disease, internal medicine, intensive care) must become a cornerstone of continuous medical education, to keep the pace with the most up‐to‐date diagnostic and therapeutic strategies [4]. From a research perspective, diabetologists should be encouraged to increase the initiation of studies, especially clinical trials, investigating pharmacological strategies to reduce mortality and burden of complications, tailored specifically to people with DFS, a high‐risk population notoriously underrepresented or excluded from major landmark cardiovascular and renal outcome studies.

Other than medical management, DFS clearly necessitates a surgical mindset to achieve limb‐salvage goals [5]. Unfortunately, traditional surgical specialties, including general, vascular, plastic, and orthopaedic surgery, frequently show limited interest in taking long‐term ownership of this specific pathology. This surgical vacuum leaves the operative and reconstructive field wide open, positioning the diabetologist to naturally step into the surgical arena; the rounded figure of the clinician‐surgeon who treats patients with an internist approach from a systemic point of view, and with an orthoplastic experience in surgical reconstruction is the key to saving the life and the limb of our patients [6].

Post‐graduate education must establish mandatory, hands‐on, surgical clerkships integrated directly into residency programs for endocrinology and diabetology. We advocate for a compulsory rotation of at least 6 months in dedicated diabetic foot surgical centres. During this intensive immersive period, residents must achieve expertise and autonomy in performing wound debridement, regenerative medicine applications, foundational orthoplastic procedures, basic osteotomies, and minor amputations. Certainly, these surgical competencies must be developed alongside the consolidation of essential diabetic foot care: prevention strategies, vascular assessment, diagnostic imaging, wound healing principles, and offloading interventions [7].

To bridge the gap between basic surgical skills and advanced limb‐salvage reconstruction, enrolment in human cadaver lab training and high‐fidelity simulation must be encouraged. Practical simulation labs provide an indispensable, risk‐free environment for acquiring mastery over basic surgical manoeuvres. Cadaveric training could be a feasible opportunity to learn local skin flap design, complex Charcot foot osteotomies, and application of external fixation devices before executing these procedures in the operating theatre under expert supervision [8].

Dedicated fellowships represent crucial turning points for cultivating clinician‐surgeons in this field. These intensive programs should offer direct exposure to live surgery of complex reconstructions, high‐volume case management, and multidisciplinary operative strategies.

However, mastering this intersection of advanced clinical medicine, targeted diagnostic imaging, cutting‐edge research innovation, and highly specialised surgical interventions requires a major change in medical education. We urgently need more dedicated, advanced training centres specifically designed to help diabetologists safely acquire clinical, diagnostic, research and surgical abilities. Without these specialised hubs to cultivate expertise in lowering the mortality risk and acquiring limb‐saving techniques also through regenerative and innovative approaches [9], care will remain unsustainably concentrated, forcing vulnerable patients into desperate ‘pilgrimages of hope’ to find adequate treatment.

Finally, to harmonise these educational interventions globally, scientific societies must establish an officially recognised, international curriculum for the DFS specialists. This standardized framework must explicitly define mandatory core competencies and elective advanced skills to be acquired by the diabetologist. Defining this clear scope of practice will standardise care equity, elevate professional recognition, and eliminate regional disparities in patient outcomes.

Furthermore, this paradigm shift in medical education must also originate within undergraduate medical school programs. It is essential to integrate mandatory hands‐on clinical modules and standardized triage protocols into undergraduate medical curricula. Every future physician must be able to recognise the clinical presentation of DFS, perform basic vascular and neurological screening, and execute proper, timely, and effective referrals to the diabetologist within structured emergency and primary care pathways.

For the modern diabetologists, managing people with DFS represents an extraordinary professional privilege, and a severe responsibility. The diabetologist is uniquely positioned to act as the primary leader of the multidisciplinary team. It is time for our profession to move past the culture of quick cross‐referrals, invest in advanced training, and finally assume complete ownership of these neglected and fragile patients.

## Author Contributions

**Vittorio Oteri:** conceptualisation, investigation, methodology, resources, writing – original draft preparation, writing – review and editing. **Luca Dalla Paola:** conceptualisation, investigation, methodology, resources, supervision, writing – review and editing. All authors whose names appear on the submission: made substantial contributions to the conception or design of the work, or the acquisition, analysis, or interpretation of data, drafted the work or revised it critically for important intellectual content, approved the version to be published and agree to be accountable for all aspects of the work in ensuring that questions related to the accuracy or integrity of any part of the work are appropriately investigated and resolved.

## Funding

The authors have nothing to report.

## Data Availability

Data sharing not applicable to this article as no datasets were generated or analysed during the current study.
